# Supplementary material for: Complexome profiling of the Chlamydomonas psb28 mutant reveals TEF5 as an early PSII assembly factor
Source: Plant Cell. 2025 Mar 17;37(6):koaf055. doi: 10.1093/plcell/koaf055 (PMC12164590; doi:10.1093/plcell/koaf055)
Supplement: koaf055_Supplementary_Data [file koaf055_supplementary_data.zip › psb28_interactive.zip!/plots/intensity/qualityPlots/peptides/SDH1_Cre14.g619133.t1.1.html]

### Cre14.g619133.t1.1

Trivial name: SDH1  
SeqCoverage [median]: 41.30  
Mass: 68.88 kDa  
  
 

| Sequence | MissedCleavage | Mass [Da] | wtIntensity | muIntensity | Score |
| --- | --- | --- | --- | --- | --- |
| AFGGQSLDFGK | 0 | 1125.55 | 58391.70 | 246211.94 | 223.31 |
| AFGGQSLDFGKGGQAYR | 1 | 1757.85 | 3887.60 | 7874.32 | 115.50 |
| AVIELENYGLPFSR | 0 | 1606.84 | 83019.85 | 114476.24 | 276.35 |
| DDANWMK | 0 | 878.36 | 813.91 | 3833.04 | 127.12 |
| DHIYLHLNHLPPELLAER | 0 | 2179.15 | 0.00 | 3727.76 | 91.48 |
| DLASRDVVSR | 1 | 1116.59 | 2716.45 | 11959.10 | 101.39 |
| EDFTQR | 0 | 794.36 | 40880.95 | 84924.94 | 166.22 |
| FQAHQTVLATGGYGR | 0 | 1604.81 | 119320.19 | 212455.99 | 201.65 |
| GAHAREDFTQR | 1 | 1286.61 | 1533.63 | 5350.86 | 93.67 |
| GEGGILR | 0 | 700.39 | 51586.62 | 136413.81 | 187.72 |
| HTLGFLPSTNDK | 0 | 1328.67 | 4977.05 | 49385.19 | 181.53 |
| IGQIMKPNTPHKPLPATAGEGAVAR | 2 | 2553.39 | 23197.45 | 47456.29 | 84.33 |
| LGANSLLDIVVFGR | 0 | 1472.84 | 9537.31 | 16130.62 | 304.57 |
| LIDETMASFQDVK | 0 | 1495.72 | 16895.72 | 42426.38 | 228.83 |
| NSEGERFMER | 1 | 1253.55 | 45687.40 | 94432.53 | 98.04 |
| SMTMEIR | 0 | 866.40 | 78605.14 | 104197.20 | 139.31 |
| SMTMEIREGR | 1 | 1208.56 | 1421.90 | 618.93 | 49.30 |
| TAEIRR | 1 | 744.42 | 5079.01 | 9916.73 | 135.50 |
| TEDGKIYQR | 1 | 1108.55 | 71553.12 | 106527.06 | 153.24 |
| TGHAMLHTLYGQAMK | 0 | 1657.81 | 14671.74 | 37054.83 | 116.58 |
| VMQNNAAVFR | 0 | 1148.58 | 133833.77 | 237166.22 | 255.54 |
| VNISYR | 0 | 750.40 | 0.00 | 1335.44 | 99.92 |
| VNISYRPVHMKPLSEEMPFIPPKPR | 3 | 2961.57 | 1551.30 | 4387.77 | 61.51 |
| WHAYDTIK | 0 | 1032.50 | 0.00 | 799.35 | 81.30 |
| YAPTAKDLASR | 1 | 1191.62 | 0.00 | 468.89 | 51.01 |
|  |  |  | 769161.8 | 1579531.4 |  |
